# The levels of systemic inflammatory markers exhibit a positive correlation with the occurrence of heart failure: a cross-sectional study from NHANES

**DOI:** 10.3389/fcvm.2024.1457534

**Published:** 2024-10-11

**Authors:** Lei Huang, Ronghuan Shen, Hailan Yu, Nake Jin, Jun Hong, Yu Luo, Xudong Chen, Jiacheng Rong

**Affiliations:** ^1^ Department of Cardiology, Ningbo Hangzhou Bay Hospital, Ningbo, China; ^2^ Department of Nursing, The First Affiliated Hospital of Ningbo University, Ningbo, China; ^3^ Department of Nursing, Ningbo Medical Center Lihuili Hospital, Ningbo, China; ^4^ Department of Intensive Care Unit, Ningbo Hangzhou Bay Hospital, Ningbo, China

**Keywords:** systemic inflammation markers, neutrophil to lymphocyte ratio, platelet to lymphocyte ratio, systemic immune inflammation index, system inflammation response index, aggregate index of systemic inflammation, heart failure

## Abstract

**Background:**

We conducted a large-scale epidemiological analysis to investigate the associations between systemic inflammation markers and heart failure (HF). Our aim is to identify potential biomarkers for early detection of HF.

**Methods:**

A cross-sectional study was conducted using data from the National Health and Nutrition Examination Survey. We investigated the associations between five systemic inflammation markers (neutrophil to lymphocyte ratio [NLR], platelet to lymphocyte ratio [PLR], systemic immune inflammation index [SII], system inflammation response index [SIRI], and aggregate index of systemic inflammation [AISI]) and the risk of HF.

**Results:**

The prevalence rates of HF exhibited a gradual increase across increasing logNLR, logPLR, logSII, logSIRI, and logAISI tertiles. Compared to those in the highest tertiles of logNLR, logSII, logSIRI, and logAISI had a 1.579-fold, 1.341-fold, 1.956-fold, and 1.499-fold increased risk of HF compared to those in the lowest tertile respectively. Conversely, there was no significant correlation between logPLR and HF risk among subjects in the highest tertile. The restricted cubic splines (RCS) analysis revealed a non-linear relationship between the elevation of systemic inflammation markers and HF prevalence. Specifically, a per standard deviation increase in any of these variables is associated with a respective 45%, 29%, 28%, 44% and 29% increase in HF prevalence. The receiver operating characteristic (ROC) analysis demonstrated favorable sensitivity and specificity of these systemic inflammation markers in detecting the presence of HF.

**Conclusion:**

Our cross-sectional study demonstrates significant positive correlations between the NLR, PLR, SII, SIRI, and AISI with the incidence of HF.

## 1 Introduction

Heart failure (HF) has emerged as a significant global health concern, resulting from structural or functional abnormalities of the heart in nearly all chronic cardiovascular diseases. It is primarily characterized by disruptions in both pulmonary and systemic circulation (1). Reaching a comprehensive comprehension of the risk factors and their intricate interrelationships with heart failure is imperative in order to explore novel avenues for prevention.

Inflammation plays a significant role in the pathogenesis of complications associated with cardiovascular diseases, including HF. Elevated levels of inflammatory markers have been demonstrated to possess prognostic value for future cardiovascular events (2). For instance, following an acute myocardial infarction, immediate activation of multiple local processes ensues, leading to the release of reactive oxygen species and cytokines. Neutrophils and monocytes migrate towards the injured tissue, thereby contributing to the initiation of acute myocardial injury (3). Neutrophils constitute the predominant population of leukocytes in humans and play a crucial role in immune responses. These cells exhibit remarkable phenotypic plasticity, which is highly influenced by the local microenvironment. In response to inflammation, neutrophils swiftly migrate towards the site of tissue damage (4). Recent studies have revealed that neutrophils are considered biomarkers of cardiovascular disease due to their crucial roles in both cardiovascular inflammation and repair (5). Bloodstream monocytes serve as precursors for dendritic cells and tissue macrophages. This particular subset of white blood cells is recruited in various infectious diseases, playing a crucial role in the systemic inflammatory response and thus contributing significantly to the pathogenesis of an aseptic inflammatory state (6, 7). The development and progression of atherosclerosis can be associated with specific chemokine receptors expressed on monocytes (6). In addition, the platelet activation in congestive heart failure (CHF) has been extensively described using various methods, and these platelet abnormalities may contribute to an increased risk of thrombosis-related complications (8, 9).

The interactions between platelets and leukocytes are increasingly recognized as crucial contributors to a persistent systemic inflammatory condition. In multiple diseases, ratios involving neutrophil and monocyte count, as well as other hematological counts such as white cell, lymphocyte, and platelet counts, could offer additional insights into the development and progression of complications (10–12). Recently, multiple studies have emphasized different markers of systemic inflammation in peripheral blood cells such as the neutrophil to lymphocyte ratio (NLR), platelet to lymphocyte ratio (PLR), systemic immune inflammation index (SII), system inflammation response index (SIRI), and aggregate index of systemic inflammation (AISI). These markers are closely associated with both cardiovascular and non-cardiovascular disorders (12, 13).

In recent years, there has been a growing interest in investigating the role of systemic inflammation and immune dysregulation in the development of HF. Despite the observed association between NLR, PLR and SII and HF (14–17), there is currently a lack of research investigating the correlation between SIRI, AISI, and HF, as well as no studies that concurrently compare these five systemic inflammation markers. Therefore, we present a comprehensive epidemiological analysis to gain deeper insights into the associations between SII, SIRI, and AISI with both HF prevalence. The primary aim of this study is to collect supporting data for potential biomarkers that could aid in the timely identification of HF.

## 2 Materials and methods

### 2.1 Study population

This study utilized data from the National Health and Nutritional Examination Survey (NHANES), which is a comprehensive database representing civilian, non-institutionalized individuals residing in the United States. The National Center for Health Statistics (NCHS) at the Centers for Disease Control and Prevention is responsible for administering the database. NHANES surveys are demographically-based, employing a complex and multistage survey design to select samples. We conducted data analysis on the most recent 12 NHANES survey cycles, which spanned from 1999 to March 2020 Pre-pandemic, and included a total of 119,664 participants. A total of 49,533 participants were included in the final analysis after excluding those younger than 20 years old (*n* = 55,351), individuals lacking information on systemic inflammatory markers (*n* = 6,539), participants with unavailable HF diagnosis (*n* = 173), and those with missing dates (*n* = 8,068).

### 2.2 Systemic inflammatory markers

According to the NHANES protocol, automated hematology analyzing devices were used to measure lymphocytes, monocytes, neutrophils and platelets count through complete blood count analysis. The systemic inflammation markers, NLR, PLR, SII, SIRI, and AISI were calculated based on the peripheral blood cell counts. NLR was calculated as follows:Neutrophil count/lymphocytes count. PLR was calculated as follows: platelets count/lymphocytes count. SII was calculated as follows: platelets count × neutrophils count/lymphocytes count. SIRI was calculated as follows: neutrophils count × monocytes count/lymphocytes count. AISI was calculated as follows: neutrophils count × platelets count × monocytescount platelets/lymphocytes count.

### 2.3 Covariates information

Our study also identified potential covariates that may impact the association between systemic inflammation markers and HF based on clinical relevance. These covariates include age, gender, race, education level, smoking status, alcohol status, body mass index (BMI), hypertension, diabetes, coronary heart disease, angina pectoris, heart attack, total cholesterol levels (TC), direct high-density lipoprotein cholesterol levels(HDL-C), low-density lipoprotein cholesterol levels(LDL-C), triglyceride levels(TG), glucose levels(GLU) and glycated hemoglobin levels(HbA1c). The HF data in NHANES were obtained through a personal interview conducted as part of a comprehensive health questionnaire. Individuals were classified as having HF if they responded affirmatively to the question “Has a doctor or other health professional ever told you that you had congestive heart failure?”. The definitions of hypertension, diabetes, coronary heart disease, angina, and heart attack were derived from data obtained through self-reported questionnaires.

### 2.4 Statistical analysis

The participants' baseline characteristics were categorized into non-HF and HF groups. Continuous variables were represented using the median (interquartile range), while categorical variables were presented as numerical values and percentages. Comparisons of statistical significance between the two groups were conducted using *χ*^2^ tests for categorical variables, one-way ANOVA tests for data with a normal distribution, or Mann–Whitney *U* tests for data without a normal distribution. The five systemic inflammation markers were analyzed as continuous independent variables and scaled per 1-unit increment in log-transformed or divided into tertiles to explore their associations with the prevalence of HF. Multivariate logistic regression models were utilized, incorporating different modifications, to calculate the odds ratios (ORs) and their corresponding 95% confidence intervals (CIs). To explore the potential non-linear relationship between HF and five markers of systemic inflammation, we utilized restricted cubic splines (RCS) analysis. In instances where the RCS analysis indicated a curve with a distinct inflection point, exhibiting either a U-shape, an inverted U-shape, or an L-shape, we divided the data into two separate segments based on this inflection point. Subsequently, segmented regression analysis was performed independently for each group. In addition, the analysis of receiver operating characteristic (ROC) curves was employed to identify the most effective threshold values for the five markers of systemic inflammation in predicting the onset of HF. A significance level of *P* < 0.05 (two-tailed) was deemed to indicate statistical significance. All the analyses were performed with R and SPSS software.

## 3 Results

### 3.1 Baseline characteristics

Table 1 presents the demographic characteristics of the study participants, including a total of 49,533 individuals aged between 20 and 85 years who were included in the analysis. Among the participants, 3.3% had HF, 48.2% were males, and the median age was 50 years old. Overall, there were significant differences in baseline characteristics between the non-HF and HF groups. The levels of NLR, PLR, SII, SIRI and AISI were significantly higher in patients with HF compared to those without HF. Non-normally distributed continuous variables were logarithmically transformed for analysis purposes. The results indicated that the difference in log-transformed NLR, PLR, SII, SIRI and AISI remained significant.

**Table 1:** Baseline characteristics of NHANES participants included in this study.

	Total (*N* = 49,533)	Non-HF (*N* = 47,886)	HF (*N* = 1,647)	*P*
Ages (years)	50.0 (34.0, 64.0)	49.0 (34.0, 64.0)	70 (61.0, 78.0)	<0.001
Gender				<0.001
Male (%)	23,878 (48.2	22,948 (47.9)	930 (56.5)	
Female (%)	25,655 (51.8)	24,938 (52.1)	717 (43.5)	
Race				<0.001
Mexican American (%)	8,231 (16.6)	8,076 (16.9)	155 (9.4)	
Other Hispanic (%)	4,347 (8.8)	4,235 (8.8)	112 (6.8)	
Non-Hispanic White (%)	21,466 (43.3)	20,582 (43.0)	884 (53.7)	
Non-Hispanic Black (%)	10,496 (21.2)	10,085 (21.1)	411 (25.0)	
Other Race (%)	4,993 (10.1)	4,908 (10.2)	85 (5.2)	
Education				<0.001
Less than high school (%)	12,326 (24.9)	11,739 (24.5)	578 (36.5)	
High school (%)	11,582 (23.4)	11,149 (23.3)	433 (26.3)	
Above high school (%)	25,625 (51.7)	24,998 (52.2)	627 (38.1)	
Smoking				<0.001
No (%)	27,376 (55.3)	26,736 (55.8)	640 (38.9)	
Yes (%)	22,157 (44.7)	21,150 (44.2)	1,007 (61.1)	
Alcohol				0.01
No (%)	12,437 (25.1)	11,979 (25.0)	458 (27.8)	
Yes (%)	37,096 (74.9)	35,907 (75.0)	1,189 (72.0)	
Hypertension				<0.001
No (%)	31,718 (64.0)	31,368 (65.5)	350 (21.3)	
Yes (%)	17,745 (35.8)	16,453 (34.4)	1,292 (78.4)	
Not recorded (%)	70 (0.1)	65 (0.1)	5 (0.3)	
Coronary heart disease				<0.001
No (%)	47,315 (95.5)	46,383 (96.9)	932 (56.6)	
Yes (%)	2,071 (4.2)	1,405 (2.9)	666 (40.4)	
Not recorded (%)	147 (0.3)	98 (0.2)	49 (3.0)	
Angina pectoris				<0.001
No (%)	48,032 (97.0)	46,820 (97.8)	1,212 (73.6)	
Yes (%)	1,370 (2.8)	972 (2.0)	398 (24.2)	
Not recorded (%)	131 (0.3)	94 (0.2)	37 (2.2)	
Heart attack				<0.001
No (%)	47,319 (95.5)	46,403 (96.9)	916 (55.6)	
Yes (%)	2,150 (4.3)	1,431 (3.0)	719 (43.7)	
Not recorded (%)	64 (0.1)	52 (0.1)	12 (0.7)	
Diabetes				<0.001
No (%)	42,000 (84.8)	41,083 (85.8)	917 (55.7)	
Yes (%)	6,384 (12.9)	5,710 (11.9)	647 (40.9)	
Borderline (%)	1,124 (2.3)	1,068 (2.2)	56 (3.4)	
Not recorded (%)	25 (0.1)	25 (0.1)	0 (0)	
BMI (kg/m^2^)	28.10 (24.45, 32.70)	28.04 (24.40, 32.60)	30.42 (26.16, 35.94)	<0.001
Lymphocyte number (1,000 cells/μl)	2.00 (1.60, 2.50)	2.10 (1.70, 2.50)	1.80 (1.40, 2.40)	<0.001
Monocyte number (1,000 cells/μl)	0.50 (0.40, 0.70)	0.50 (0.40, 0.70)	0.60 (0.50, 0.70)	<0.001
Neutrophils number (1,000 cells/μl)	4.00 (3.10, 5.20)	4.00 (3.10, 5.10)	4.50 (3.50, 5.60)	<0.001
Platelet count (1,000 cells/μl)	243.00 (206.00, 287.00)	244.00 (206.00, 287.00)	218.00 (180.00, 265.00)	0.683
TC (mmol/L)	4.94 (4.27, 5.69)	4.94 (4.27, 5.69)	4.42 (3.72, 5.33)	0.002
TG (mmol/L)	1.36 (0.91, 2.06)	1.34 (0.90, 2.06)	1.54 (1.06, 2.24)	<0.001
LDL-C (mmol/L)	2.87 (2.30, 3.51)	2.89 (2.32, 3.52)	2.45 (1.84, 3.20)	<0.001
HDL-C (mmol/L)	1.31 (1.09, 1.60)	1.32 (1.09, 1.60)	1.19 (0.99, 1.47)	<0.001
GLU (mmol/L)	5.11 (4.72, 5.72)	5.11 (4.72, 5.72)	5.66 (5.11, 7.11)	<0.001
HbA1c (%)	5.50 (5.20, 5.90)	5.50 (5.20, 5.80)	5.90 (5.50, 6.70)	<0.001
NLR	1.95 (1.46, 2.62)	1.94 (1.45, 2.60)	2.42 (1.68, 3.36)	<0.001
PLR	118.93 (94.12, 150.48)	118.95 (94.21, 150.00)	118.24 (91.25, 157.50)	0.683
SII	474.16 (336.36, 671.46)	472.50 (335.76, 668.06)	522.67 (353.70, 771.43)	<0.001
SIRI	1.04 (0.70, 1.54)	1.03 (0.70, 1.52)	1.47 (0.95, 2.25)	<0.001
AISI	251.54 (161.78, 392.13)	249.60 (160.78, 388.57)	319.20 (195.88, 504.81)	<0.001
LogNLR	0.29 (0.16, 0.42)	0.29 (0.16, 0.42)	0.38 (0.23, 0.53)	<0.001
LogPLR	2.08 (1.97, 2.18)	2.08 (1.97, 2.18)	2.07 (1.96, 2.20)	0.683
LogSII	2.68 (2.53, 2.83)	2.67 (2.53, 2.82)	2.72 (2.55, 2.89)	
LogSIRI	0.02 (−0.15, 0.19)	0.01 (−0.16, 0.18)	0.17 (−0.02, 0.35)	<0.001
LogAISI	2.40 (2.21, 2.59)	2.40 (2.21, 2.59)	2.50 (2.29, 2.70)	<0.001

BMI, body mass index; TC, total cholesterol; TG, triglyceride; LDL-C, low density lipoprotein cholesterol; HDL-C, high density lipoprotein cholesterol; GLU, glucose; HbA1c, glycated hemoglobin; NLR, neutrophil to lymphocyte ratio; PLR, platelet to lymphocyte ratio; SII, systemic immune inflammation index; SIRI, system inflammation response index; AISI, aggregate index of systemic inflammation.

### 3.2 Systemic inflammation markers and HF proportion

**Figure 1:**
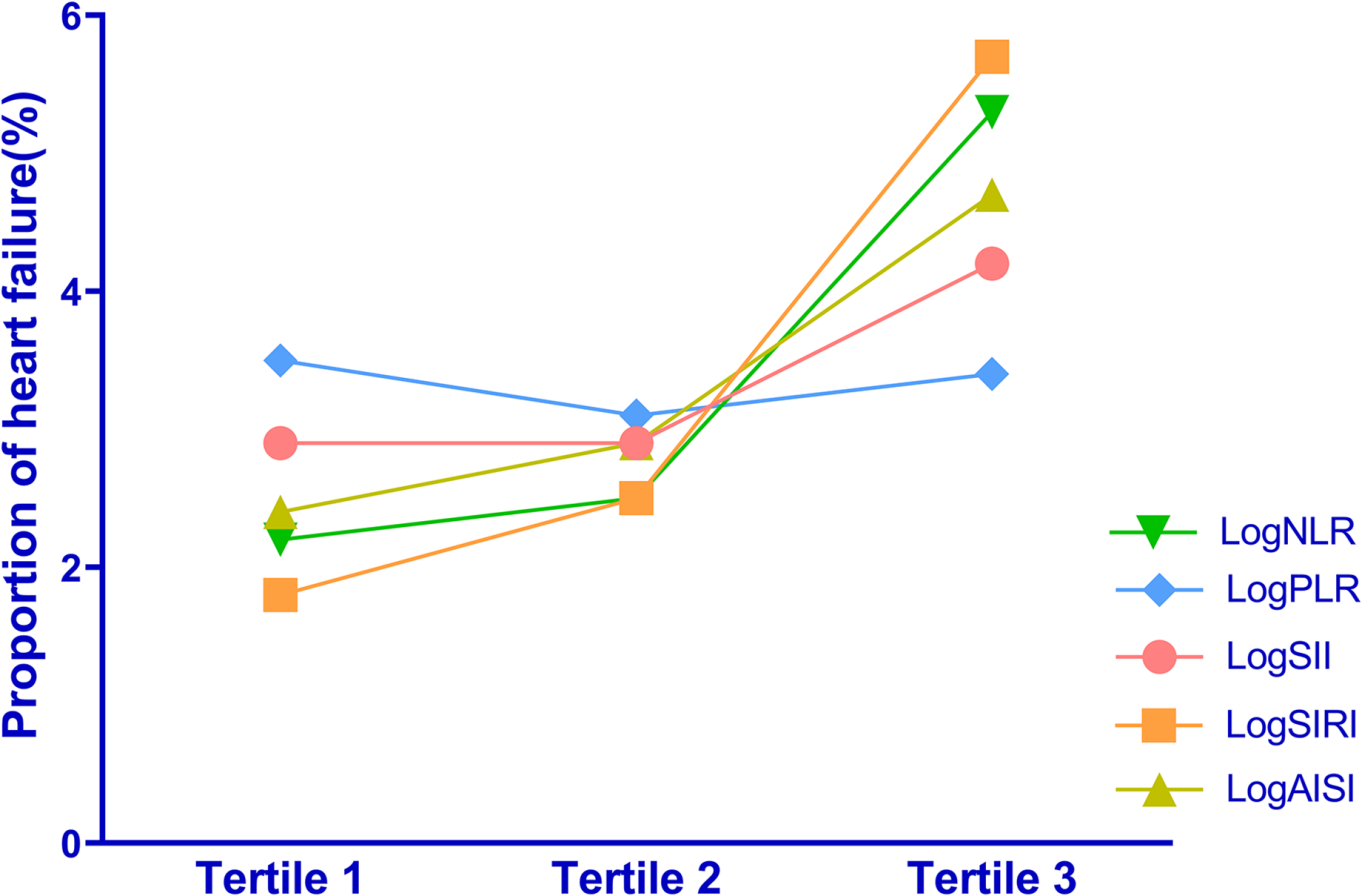
Distribution of HF proportions among different tertiles of the systemic inflammation markers.

To explore the potential association between systemic inflammation markers and the proportion of HF, we performed additional analyses by dividing participants into three tertiles based on their log-transformed levels (Refer to Supplementary Table S1 for a detailed breakdown of the grouping). Our study investigated the proportion of HF across tertiles of logNLR, logPLR, logSII, logSIRI and logAISI. In logNLR, the number of patients with HF in tertiles 1–3 were 365, 414 and 868, respectively, corresponding to proportions of 2.2%, 2.5%, and 5.3%. In logPLR, the number of patients with HF in tertiles 1–3 were 570, 514 and 565, respectively, with proportion of 3.5%, 3.1% and 3.4%. For the logSII group, the number of patients with HF in tertiles was as follows: tertile 1 had a count of 483; tertile 2 had a count of 472; and tertile 3 had a count of 692. The corresponding proportions increased from tertile one to three (2.9%, 2.9%, and 4.2% respectively). In terms of logSIRI, the number of patients with HF in tertiles 1–3 were 293, 417 and 937, respectively, with proportion of 1.8%, 2.5% and 5.7%. Similarly, in logAISI, the number of patients with HF in tertiles 1–3 were 390, 475, and 728, respectively, with proportion of 2.4%, 2.9% and 4.7%. Overall, these results demonstrate a gradual escalation in the proportion of HF as logNLR, logPLR, logSII, logSIRI and logAISI tertiles increase (Figure 1).

### 3.3 Systemic inflammation markers and HF risk

**Figure 2:**
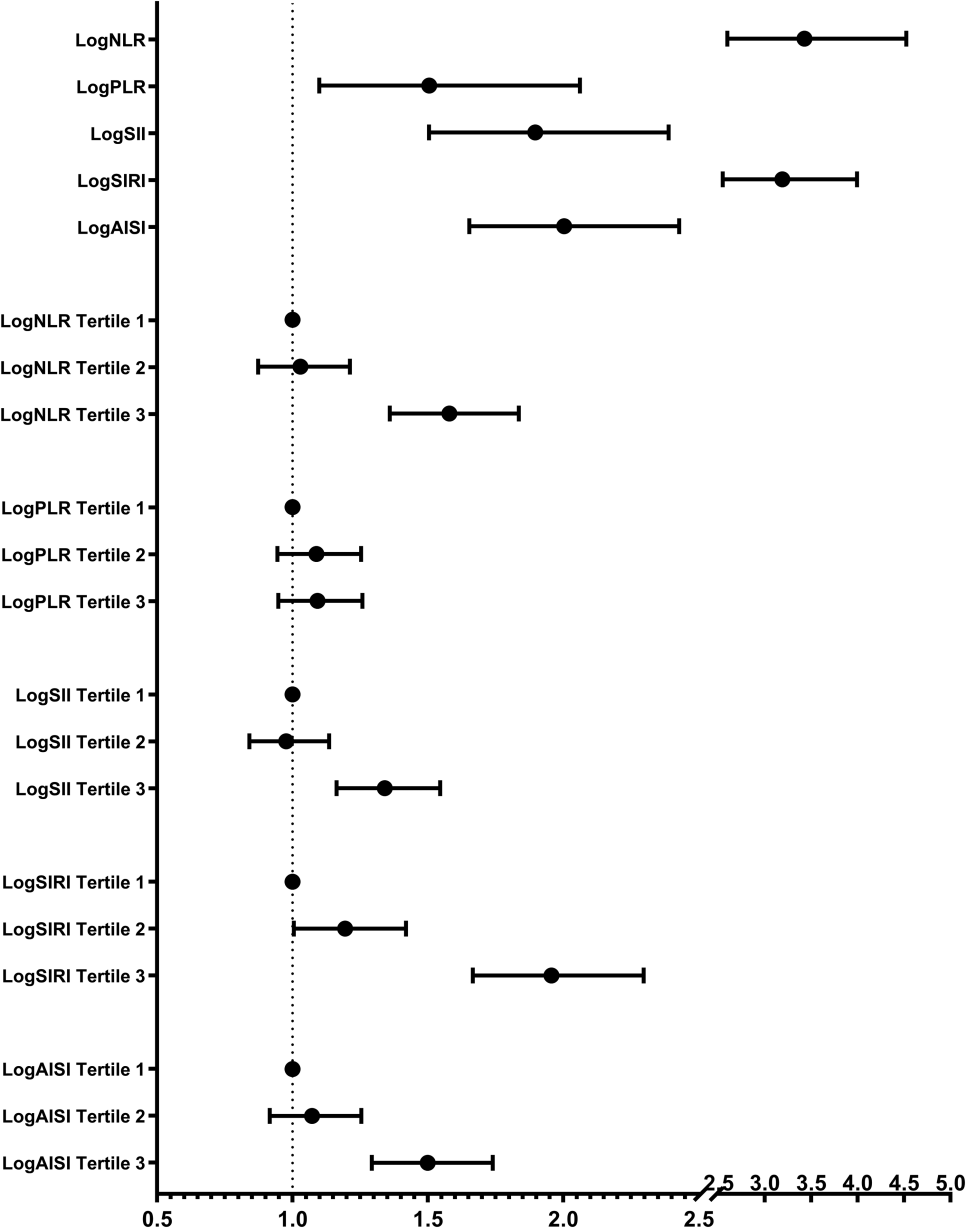
Multivariate-adjusted OR (95% CI) of the relationships between the systemic inflammation markers and HF prevalence in continuous and tertiles analyses. Adjusted for gender, age, race, education, smoking, alcohol, hypertension, coronary heart disease, angina pectoris, heart attack, diabetes, body mass index, total cholesterol, triglyceride, low density lipoprotein cholesterol, high density lipoprotein cholesterol, glucose, and glycated hemoglobin.

**Figure 3:**
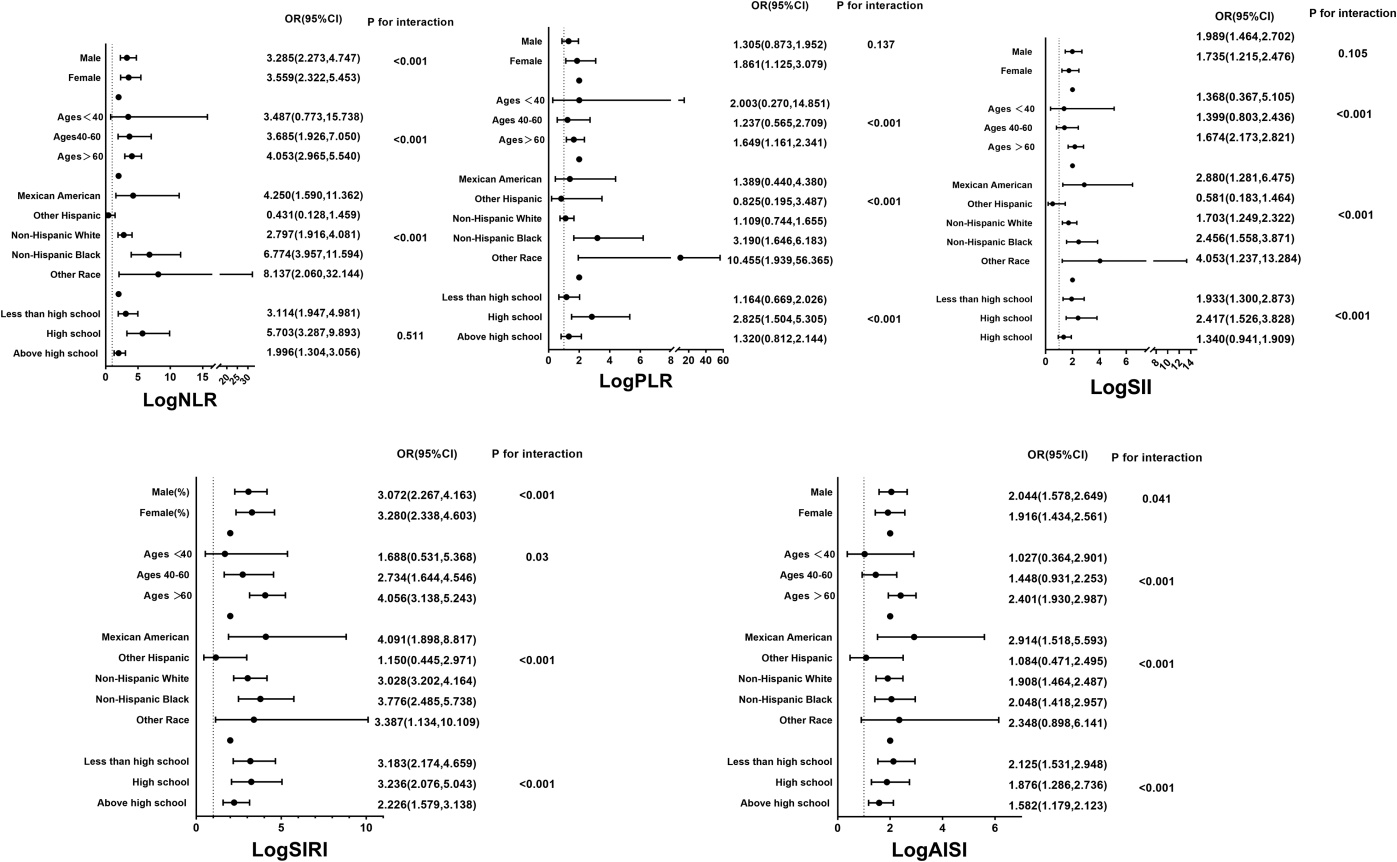
Subgroup analyses for the associations between systemic inflammation markers and the prevalence of HF stratified by participant characteristics. Results are expressed as multivariable-adjusted OR in continuous analyses after controlling covariates including gender, age, race, education, smoking, alcohol, hypertension, coronary heart disease, angina pectoris, heart attack, diabetes, body mass index, total cholesterol, triglyceride, low density lipoprotein cholesterol, high density lipoprotein cholesterol, glucose, and glycated hemoglobin.

The results of the multivariate logistic regression analysis demonstrate a significant association between elevated tertiles of the systemic inflammation markers and an increased risk of HF (Table 2). These associations of logNLR, logSII, logSIRI and logAISI are significant in both the unadjusted model and the partially adjusted model. From the fully adjusted model, compared to those in the lowest tertile, individuals in the highest logNLR, logSII, logSIRI and logAISI tertiles exhibited a 1.579-fold, 1.341-flod, 1.956-flod and 1.499-fold increased risks of HF, respectively. In contrast, we did not observe a significant correlation between logPLR and HF risk among subjects in the highest tertile of logPLR (Table 2 and Figure 2). To assess the consistency of the association between these systemic inflammation markers and HF across various subgroups, a subgroup analysis was conducted (Figure 3 and Supplementary Table S2). These findings indicate that the relationship between systemic inflammation markers and HF remains consistent across different subgroups, demonstrating high stability and reliability.

**Table 2:** Associations between three systemic inflammation markers and HF risk.

	Model 1		Model 2		Model 3	
	OR (95% CI)	*P*	OR (95% CI)	*P*	OR (95% CI)	*P*
LogNLR	9.072 (7.209, 11.416)	<0.001	3.623 (2.785,4.714)	<0.001	3.427 (2.596, 4.525)	<0.001
LogNLR categories						
T1	Reference		Reference		Reference	
T2	1.132 (0.982, 1.306)	0.087	1.059 (0.902,1.243)	0.485	1.029 (0.873, 1.212)	0.734
T3	2.466 (2.178, 2.792)	<0.001	1.689 (1.460,1.955)	<0.001	1.579 (1.359, 1.836)	<0.001
*P* for trend	<0.001		<0.001		<0.001	
LogPLR	1.212 (0.902, 1.629)	0.201	1.358 (1.009, 1.827)	0.043	1.505 (1.099, 2.061)	0.011
LogPLR categories						
T1	Reference		Reference		Reference	
T2	0.900 (0.797, 1.016)	0.088	1.056 (0.919, 1.213)	0.443	1.088 (0.944, 1.253)	0.247
T3	0.987 (0.877, 1.112)	0.835	1.045 (0.912, 1.198)	0.523	1.092 (0.948, 1.258)	0.225
*P* for trend	<0.001		0.52		0.22	
LogSII	2.344 (1.908, 2.880)	<0.001	1.980 (1.585, 2.474)	<0.001	1.896 (1.504, 2.389)	<0.001
LogSII categories						
T1	Reference		Reference		Reference	
T2	0.976 (0.858, 1.111)	0.717	0.991 (0.856, 1.147)	0.903	0.977 (0.841, 1.135)	0.762
T3	1.451 (1.289, 1.633)	<0.001	1.395 (1.215, 1.601)	<0.001	1.341 (1.163, 1.545)	<0.001
*P* for trend	<0.001		<0.001		<0.001	
LogSIRI	7.665 (6.401, 9.179)	<0.001	3.444 (2.774, 4.275)	<0.001	3.188 (2.545, 3.993)	<0.001
LogSIRI categories						
T 1	Reference		Reference		Reference	
T 2	1.432 (1.231, 1.665)	<0.001	1.229 (1.038, 1.454)	0.017	1.194 (1.005, 1.419)	0.044
T 3	3.328 (2.913, 3.801)	<0.001	2.097 (1.795,2.450)	<0.001	1.956 (1.666, 2.297)	<0.001
*P* for trend	<0.001		<0.001		<0.001	
LogAISI	3.048 (2.590, 3.588)	<0.001	2.114 (1.755, 2.546)	<0.001	2.003 (1.653, 2.428)	<0.001
LogAISI categories						
T 1	Reference		Reference		Reference	
T 2	1,224 (1.069, 1.402)	0.003	1.112 (0.954,1.296)	0.174	1.072 (0.916, 1.254)	0.387
T 3	2.054 (1.816, 2.324)	<0.001	580 (1.368, 1.825)	<0.001	1.499 (1.293, 1.739)	<0.001
*P* for trend	<0.001		<0.001		<0.001	

OR, odds ratio; CI, confidence interval.

Model 1 was not adjusted for any confounders.

Model 2 was adjusted for gender, age, race, education, smoking, alcohol, hypertension, coronary heart disease, angina pectoris, heart attack, and diabetes.

Model 3 was adjusted for gender, age, race, education, smoking, alcohol, hypertension, coronary heart disease, angina pectoris, heart attack, diabetes, body mass index, total cholesterol, triglyceride, low density lipoprotein cholesterol, high density lipoprotein cholesterol, glucose, and glycated hemoglobin.

**Figure 4:**
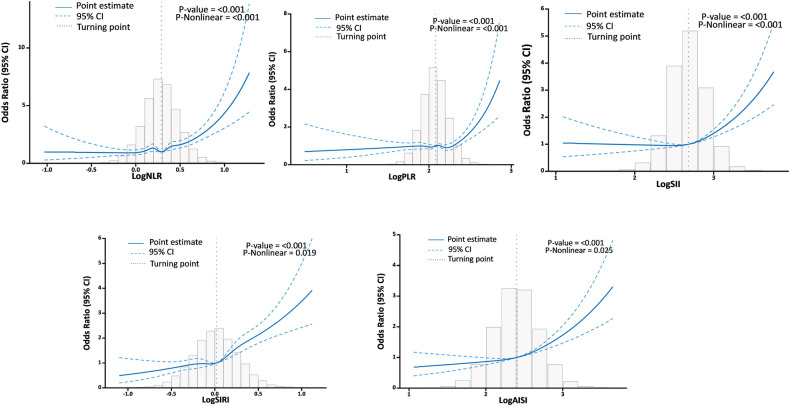
Association between systemic inflammation markers and HF with the RCS function.

The RCS analysis found a U-shaped relationship between logNLR, logPLR, logSII, logSIRI, and logAISI and HF after adjusting for various factors. The inflection point was identified at logNLR = 0.29, logPLR = 2.08, logSII = 2.68, logSIRI = 0.02, and logAISI = 2.4 (Figure 4). By utilizing the inflection point, the data was stratified into two distinct groups. Subsequently, segmented regression analysis was conducted on each group separately. When logNLR is greater than or equal to 0.29, logPLR is greater than or equal to 2.08, logSII is greater than or equal to 2.68, logSIRI is greater than or equal to 0.02 and logAISI is greater than or equal to 2.4, there exists a significant association between an increase in any of these variables by one standard deviation and a respective prevalence increase in HF by 45% (OR = 1.14; 95% CI, 1.38–1.52), 29% (OR = 1.29; 95% CI, 1.21–1.36), 28% (OR = 1.28; 95% CI, 1.21–1.35), 44% (OR = 1.44; 95% CI, l.37–l.51) and 29% (OR = l.29; 9S% CI, l.23–l.36). The results of two piecewise linear regression models are demonstrated in Table 3.

**Table 3:** Effect of standardized systemic inflammation markers on HF: adjusted odds ratios from segmented logistic regression analysis.

	OR per SD	95% CI	*P*-value
logNLR (<0.29)	1.02	0.94, 1.12	0.65
logNLR (≥0.29)	1.45	1.38, 1.52	<0.001
logPLR (<2.08)	0.89	0.85, 0.94	<0.001
logPLR (≥2.08)	1.29	1.21, 1.36	<0.001
logSII (<2.68)	0.92	0.86, 0.99	0.020
logSII (≥2.68)	1.28	1.21, 1.35	<0.001
logSIRI (<0.02)	1.11	1.01, 1.23	0.028
logSIRI (≥0.02)	1.44	1.37, 1.51	<0.001
logAISI (<2.4)	1.03	0.95, 1.12	0.48
logAISI (≥2.4)	1.29	1.23, 1.36	<0.001

OR, odds ratio; CI, confidence interval.

ORs were adjusted for gender, age, race,education ,smoking,alcohol,hypertension,coronary heart disease,angina pectoris,heart attack, diabetes, body mass index, total cholesterol, triglyceride, low density lipoprotein cholesterol, high density lipoprotein cholesterol, glucose, and glycated hemoglobin.

### 3.4 Value of systemic inflammation markers in predicting HF by ROC

**Figure 5:**
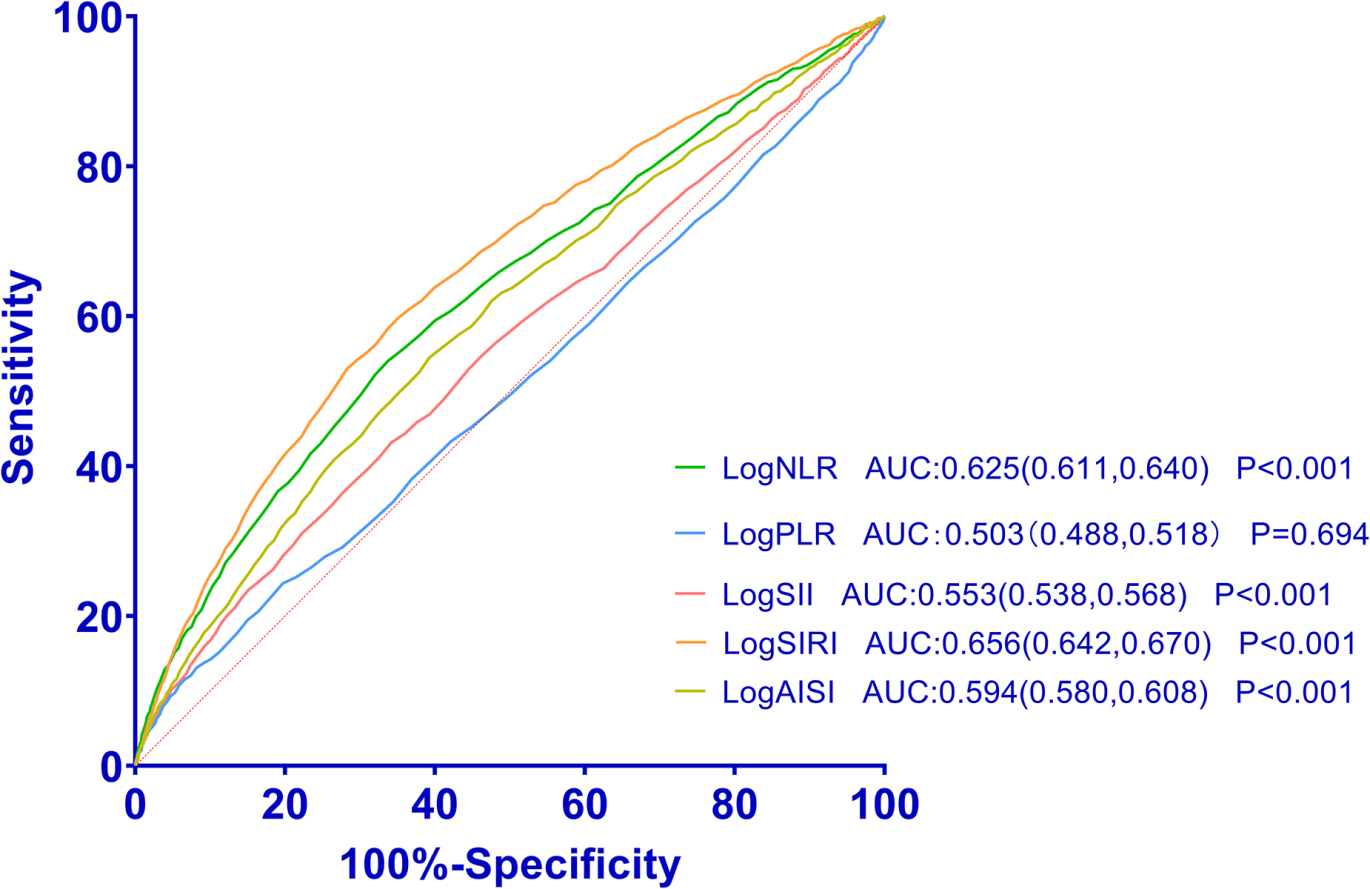
Receiver operating characteristic curves for systemic inflammation markers as a predictor of HF.

We utilized ROC curves to demonstrate the efficacy of logNLR, logPLR, logSII, logSIRI, and logAISI in distinguishing between individuals with HF and those without HF. ROC curve analysis of logNLR, logPLR, logSII, logSIRI, and logAISI showed that they had AUC of 0.625, 0.503, 0.553, 0.656 and 0.594. The ability of these systemic inflammation markers to predict HF as shown in Figure 5. The cutoff points for logNLR, logPLR, logSII, logSIRI, and logAISI were determined as 0.3, 2.07, 2.68, 0.02, and 2.41 respectively to estimate the presence of HF with a sensitivity of 65.8%, 50.5%, 57.9%, 70.1% and 63.1% as well as a specificity of 51.6%, 50.1%, 50.1%, 50.5% and 51.1%, respectively.

## 4 Discussion

In this study, we conducted a comprehensive evaluation of various systemic inflammation markers in relation to the risk of HF. The main findings of our study can be summarized as follows: (1) HF patients exhibited significantly elevated levels of NLR, PLR, SII, SIRI, and AISI compared to those without HF. Moreover, the prevalence rates of HF gradually increased with higher logNLR, logPLR, logSII, logSIRI, and logAISI tertiles. (2) Logistic regression analysis revealed a positive association between elevated levels of these systemic inflammation markers and an increased risk of HF. (3) RCS analysis demonstrated a non-linear relationship between the elevation of these systemic inflammation markers and the risk of HF when logNLR is ≥0.29, logPLR is ≥2.08, logSII is ≥2.68, logSIRI is ≥0.02, andlogAISIis ≥ 2.4. (4) ROC curve analysis showed that these systemic inflammation markers have favorable sensitivity and specificity in detecting the presence of HF.

This study is the first to assess the relationship between NLR, PLR, SII, SIRI, and AISI with HF risk using a large sample size. One of our major findings aligns with previous research conducted by Zheng et al., indicating that SII serves as a significant predictor for HF prevalence (17). In contrast to prior studies, we have utilized RCS analysis to investigate the non-linear correlation between these five systemic inflammatory markers and HF risks. Additionally, the ROC analysis was employed to investigate the predictive performance of these indicators for HF in our study. Notably, Zheng et al. did not employ RCS and ROC analysis in their assessment of the relationship between SII and HF. Similarly, unlike our study, several other investigations have not examined the correlation between multiple systemic inflammatory markers and heart failure comprehensively, as they have only investigated a limited number of these markers (14–16). The area under the receiver ROC curve, known as the AUC, is utilized in clinical studies to assess the performance of a diagnostic test or predictive model (18, 19). In our study, the AUC values ranging from 0.503 to 0.656 indeed indicate limited discriminatory power. This finding suggests that while these markers may not be strong standalone predictors, they could still have value when used in combination with other clinical indicators or risk factors. The AUC values observed in our study were comparable to those reported in other clinical studies (20). To address the potential limitations of these markers when used independently, we propose that the combined effects of these indicators on HF should be taken into consideration in clinical practice.

HF is a complicated systemic disorder that encompasses not only cardiac dysfunction but also involves intricate interplay with factors such as immune system activation and inflammation. The inflammatory response plays a crucial role in HF, encompassing the infiltration of inflammatory cells, secretion and regulation of cytokines, modulation of other cellular functions, and remodeling of the myocardial extracellular matrix (21, 22). The close association between these inflammatory responses and cardiovascular disease has made them a key focus in research on HF. The main cytokines involved in the inflammatory response of these patients are tumor necrosis factor-α (TNF-α) and interleukin-6 (IL-6), whose elevation is closely associated with the unfavorable prognosis of individuals with HF (23, 24). In addition to TNF-α and IL-6, several other inflammatory factors play pivotal roles in HF. For instance, interleukin-1β (IL-1β), interleukin-8 (IL-8), and interleukin-18 (IL-18) exert significant influences (25). These inflammatory mediators not only directly impact the survival and functionality of cardiomyocytes but also intricately contribute to the progression of HF by modulating the inflammatory response, oxidative stress, and fibrosis within the cardiovascular system (17). While the diagnosis and prognostic assessment of HF greatly benefit from these indicators, their utilization is hindered by drawbacks such as lengthy testing duration and elevated expenses. Hence, the identification of alternative biomarkers holds significant implications for enhancing HF diagnosis and treatment. These systemic inflammatory markers are comprehensive biomarkers that reflect the body's inflammatory state. They offer advantages such as simplicity, rapidity, cost-effectiveness and reliability. Compared to other biomarkers, these indicators have a wider range of applications and can effectively identify high-risk patients, guide individualized treatment plans and improve patient prognosis. Therefore, these systemic inflammatory markers hold great potential for application in the diagnosis and treatment of HF.

The findings of several studies have demonstrated a significant correlation between elevated white blood cell and neutrophil counts, indicating an augmented risk of cardiovascular events (26, 27). Neutrophils play a critical role in the body's response to inflammation, including their ability to engulf and break down bacteria and viruses. They also release enzymes and oxidants to eliminate pathogens (28). However, in individuals with HF, neutrophils can become activated and release excessive amounts of pro-inflammatory cytokines and oxidative stress substances. This activation may contribute to the progression of cardiovascular disease and the development of HF (29). On the other hand, lymphocytes are vital cells that regulate immune responses. The quantity and function of lymphocytes are closely linked to the occurrence and development of HF. A reduced lymphocyte count in HF patients is considered an indicator of systemic inflammation severity as well as prognosis (30). Lymphocytes help regulate inflammatory responses by reducing cytokine release while inhibiting activation of neutrophils and monocytes, thereby providing protection against heart injury (31). Additionally, platelets also have a significant impact on the occurrence and development of HF due to their involvement in activation and coagulation processes associated with increased incidence of cardiovascular events among patients with this condition (32). Activated platelets can contribute to inflammatory responses by releasing pro-inflammatory cytokines while interacting with other cell types such as neutrophils, monocytes, or endothelial cells (33). These systemic inflammatory markers are comprehensive new inflammatory biomarkers based on lymphocyte, neutrophil and platelet counts. By quantifying one or a combination of these inflammatory and immune markers, it can offer novel clinical evidence for the diagnosis and prognosis of HF.

Compared to NLR, SII, SIRI, and AISI, the prognostic significance of PLR on HF outcomes is relatively limited. However, the literature presents inconsistent findings in this regard. Ye et al. reported that a higher PLR was associated with unfavorable clinical outcomes in patients with acute HF and could potentially serve as a novel marker for acute HF management (32). Another recent study found that PLR independently correlated with a 3-fold increase in 30-day mortality risk following emergency department admission for acute decompensated HF (34). Conversely, Cheng et al. observed no statistically significant difference in the levels of PLR between the non-HF and HF groups (*P* > 0.05) (35). Similarly to the aforementioned study, other studies have also reported that PLR does not independently predict the prognosis of acute HF (36, 37). Consistent with several of these cited studies, our results suggest that after adjusting for relevant demographic factors and subjects’ medical history, PLR does not emerge as an independent predictor for long-term mortality in individuals with HF.

In addition, we found that there were significant differences observed in BMI, GLU, HDL-C, and TG levels between patients with and without heart failure. These findings suggest the presence of insulin resistance(IR) in patients with HF. Previous studies have consistently demonstrated that IR plays a crucial role in the pathogenesis and progression of HF (38, 39). The triglyceride glucose (TyG) index and triglyceride-to-high-density lipoprotein cholesterol (TG/HDL-C) ratio are widely recognized as simple non-insulin-based indices for assessing insulin resistance. A recent study investigated the association between the TyG index and HF risk among adults using data from 2007 to 2018 NHANES survey. The results revealed a positive correlation between the TyG index and HF risk after adjusting for potential confounders. Notably, there was also an evident “J-shaped” dose-response relationship observed between the TyG index and HF risk (40). Similarly, another cross-sectional study also indicated a strong association between elevated TyG index and TG/HDL-C ratio with the prevalence of HF in overweight/obese adults without diabetes, suggesting a nonlinear relationship (41). IR is increasingly recognized as a chronic, low-grade inflammatory state that has the potential to cause damage to cardiac tissues. Prolonged inflammation can contribute to fibrosis and remodeling of the heart, thereby further exacerbating the progression of heart failure (42).

Our study has certain inherent limitations. Firstly, the cross-sectional nature of our research prevents us from establishing any causal associations between these markers and HF prevalence. Additionally, the unavailability of follow-up peripheral blood cell counts hinders our ability to evaluate the influence of individual inflammation ratios on HF risk. Third, the study exclusively utilized samples from the U.S. populations, thus limiting the generalizability of the findings to non-U.S. Fourth, despite our best efforts to adjust for potential confounding factors, there may still be some that have influenced our results. Finally, we recognize that secondary analyses, while valuable for generating new insights, carry risks related to data integrity, selection bias, and the possibility of over-interpretation. Therefore, caution should be exercised when interpreting these findings in clinical practice.

## 5 Conclusions

In conclusion, the prevalence rates of HF gradually increased with higher logNLR, logPLR, logSII, logSIRI, and logAISI tertiles. The HF risks for individuals in the highest tertiles were elevated compared to those in the lowest tertiles. The RCS analysis revealed a non-linear relationship between the elevation of systemic inflammation markers and HF prevalence. ROC curve analysis demonstrated that these systemic inflammation markers exhibit favorable sensitivity and specificity in detecting the presence of HF. Our study suggests that these novel systemic inflammatory markers can provide a simple and reliable method to assess HF risk in individuals with varying levels of inflammation.

## Data Availability

The datasets generated and/or analysed during the current study are available from the U.S. Department of Health and Human Services, Centers for Disease Control and Prevention [https://wwwn.cdc.gov/nchs/nhanes].
