# Supplementary material for: The levels of systemic inflammatory markers exhibit a positive correlation with the occurrence of heart failure: a cross-sectional study from NHANES
Source: Front Cardiovasc Med. 2024 Oct 11;11:1457534. doi: 10.3389/fcvm.2024.1457534 (PMC11502476; doi:10.3389/fcvm.2024.1457534)
Supplement: Supplementary file 1 [file Datasheet1.docx]

| Supplementary Table 1 Systemic inflammation markers and HF prevalence | | | | | |
| --- | --- | --- | --- | --- | --- |
|  |  | Non-HF  (n) | HF  (n) | HF  Prevalence(%) | P |
| LogNLR |  |  |  |  | 0.007 |
|  | Tertile1（≤0.21） | 16146 | 365 | 2.2 |  |
|  | Tertile2（0.22-0.37） | 16171 | 414 | 2.5 |  |
|  | Tertile3（≥0.38） | 15569 | 868 | 5.3 |  |
| LogPLR |  |  |  |  | 0.182 |
|  | Tertile1（≤2.01） | 15950 | 570 | 3.5 |  |
|  | Tertile2（2.02-2.14） | 15982 | 514 | 3.1 |  |
|  | Tertile3（≥2.15） | 15954 | 563 | 3.4 |  |
| LogSII |  |  |  |  | ＜0.001 |
|  | Tertile1（≤2.58） | 16025 | 483 | 2.9 |  |
|  | Tertile2（2.59-2.77） | 16038 | 472 | 2.9 |  |
|  | Tertile3（≥2.78） | 15823 | 692 | 4.2 |  |
| LogSIRI |  |  |  |  | ＜0.001 |
|  | Tertile1（≤-0.09） | 16025 | 293 | 1.8 |  |
|  | Tertile2（-0.08—0.12） | 16027 | 417 | 2.5 |  |
|  | Tertile3（≥0.13） | 15574 | 937 | 5.7 |  |
| LogSIRI |  |  |  |  | ＜0.001 |
|  | Tertile1（≤2.28） | 16118 | 390 | 2.4 |  |
|  | Tertile2（2.29-2.52） | 16035 | 475 | 2.9 |  |
|  | Tertile3（≥2.53） | 15733 | 728 | 4.7 |  |

| Supplementary Table 2 Subgroup analyses for the associations between four systemic inflammation markers and HF risk stratified by participant characteristics in continuous analyses | | | | | | | | | | | | | | | | | | | | | | | | | | |
| --- | --- | --- | --- | --- | --- | --- | --- | --- | --- | --- | --- | --- | --- | --- | --- | --- | --- | --- | --- | --- | --- | --- | --- | --- | --- | --- |
|  |  | LogNLR |  |  | LogPLR | |  | |  | | LogSII | | | |  | | LogSIRI | | | |  | | LogAISI | | | |
|  |  | OR(95%CI) | P for interaction |  | | OR(95%CI) | | P for interaction | |  | | OR(95%CI) | | P for interaction | |  | | OR(95%CI) | | P for interaction | |  | | OR(95%CI) | P for interaction |  |
|  |  |  |  |  |  | |  | |  | |  | |  | |  | |  | |  | |  | |  | |  | |
| Male |  | 3.285(2.273,4.747) | <0.001 |  | 1.305(0.873,1.952) | | 0.137 | |  | | 1.989(1.464,2.702) | | 0.105 | |  | | 3.072(2.267,4.163) | | <0.001 | |  | | 2.044(1.578,2.649) | | 0.041 | |
| Female |  | 3.559(2.322,5.453) |  |  | 1.861(1.125,3.079) | |  |  |  | | 1.735(1.215,2.476) | |  |  |  | | 3.280(2.338,4.603) | |  |  |  | | 1.916(1.434,2.561) | |  |  |
|  |  |  |  |  |  | |  | |  | |  | |  | |  | |  | |  | |  | |  | |  | |
| Ages 20-39 |  | 3.487(0.773,15.738) | <0.001 |  | 2.003(0.270,14.851) | | <0.001 | |  | | 1.368(0.367,5.105) | | <0.001 | |  | | 1.688(0.531,5.368) | | 0.03 | |  | | 1.027(0.364,2.901) | | <0.001 | |
| Ages 40-59 |  | 3.685(1.926,7.050) |  |  | 1.237(0.565,2.709) | |  | |  | | 1.399(0.803,2.436) | |  |  |  | | 2.734(1.644,4.546) | |  |  |  | | 1.448(0.931,2.253) | |  |  |
| Ages 60+ |  | 4.053(2.965,5.540) |  |  | 1.649(1.161,2.341) | |  | |  | | 1.674(2.173,2.821) | |  |  |  | | 4.056(3.138,5.243) | |  |  |  | | 2.401(1.930,2.987) | |  |  |
|  |  |  |  |  |  | |  | |  | |  | |  | |  | |  | |  | |  | |  | |  | |
| Mexican American |  | 4.250(1.590,11.362) | <0.001 |  | 1.389(0.440,4.380) | | <0.001 | |  | | 2.880(1.281,6.475) | | <0.001 | |  | | 4.091(1.898,8.817) | | <0.001 | |  | | 2.914(1.518,5.593) | | <0.001 | |
| Other Hispanic |  | 0.431(0.128,1.459) |  |  | 0.825(0.195,3.487) | |  |  |  | | 0.581(0.183,1.464) | |  |  |  | | 1.150(0.445,2.971) | |  |  |  | | 1.084(0.471,2.495) | |  |  |
| Non-Hispanic White |  | 2.797(1.916,4.081) |  |  | 1.109(0.744,1.655) | |  |  |  | | 1.703(1.249,2.322) | |  |  |  | | 3.028(3.202,4.164) | |  |  |  | | 1.908(1.464,2.487) | |  |  |
| Non-Hispanic Black |  | 6.774(3.957,11.594) |  |  | 3.190(1.646,6.183) | |  |  |  | | 2.456(1.558,3.871) | |  |  |  | | 3.776(2.485,5.738) | |  |  |  | | 2.048(1.418,2.957) | |  |  |
| Other Race |  | 8.137(2.060,32.144) |  |  | 10.455(1.939,56.365) | |  |  |  | | 4.053(1.237,13.284) | |  |  |  | | 3.387(1.134,10.109) | |  |  |  | | 2.348(0.898,6.141) | |  |  |
|  |  |  |  |  |  | |  | |  | |  | |  | |  | |  | |  | |  | |  | |  | |
| Less than high school |  | 3.114(1.947,4.981) | 0.511 |  | 1.164(0.669,2.026) | | <0.001 | |  | | 1.933(1.300,2.873) | | <0.001 | |  | | 3.183(2.174,4.659) | | <0.001 | |  | | 2.125(1.531,2.948) | | <0.001 | |
| High school |  | 5.703(3.287,9.893) |  |  | 2.825(1.504,5.305) | |  |  |  | | 2.417(1.526,3.828) | |  |  |  | | 3.236(2.076,5.043) | |  |  |  | | 1.876(1.286,2.736) | |  |  |
| Above high school |  | 1.996(1.304,3.056) |  |  | 1.320(0.812,2.144) | |  |  |  | | 1.340(0.941,1.909) | |  |  |  | | 2.226(1.579,3.138) | |  |  |  | | 1.582(1.179,2.123) | |  |  |
